# Compensatory expression regulation of highly homologous proteins HNRNPA1 and HNRNPA2

**DOI:** 10.3906/biy-2010-29

**Published:** 2021-04-20

**Authors:** Yan CHANG, Xiaofeng LU, Jiaying QIU

**Affiliations:** 1 School of Life Sciences, Nantong University, Nantong, Jiangsu China; 2 Department of Prenatal Screening and Diagnosis Center, Affiliated Maternity and Child Health Care Hospital of Nantong University, Nantong, Jiangsu China

**Keywords:** HNRNPA1, HNRNPA2, posttranscriptional gene regulation, 3′UTR, alternative splicing

## Abstract

Heterogeneous nuclear ribonucleoprotein (HNRNP) A1 and A2 are the most abundant HNRNPs with nearly identical functions, and play important roles in regulating gene expression at multiple levels (i.e. transcription, posttranscription, and translation). However, the expression and regulation mechanism of HNRNPA1 and A2 themselves remain unclear. In this study, the amino acid sequences of HNRNPA1 and HNRNPA2 were compared and found to have 78% and 86% homology in key functional domains. Transfection of HEK293 cells with small interfering RNA and overexpression vectors of HNRNPA1 and HNRNPA2 demonstrated that HNRNPA1 and HNRNPA2 paralogs regulate each other’s expression in a compensatory manner at both the RNA and protein levels. Multiprimer reverse transcription-polymerase chain reaction showed that HNRNPA1 and HNRNPA2 did not affect splicing of the HNRNPA2 and HNRNPA1 gene. Using luciferase reporting system, we found that compensatory degradation was mediated by the 3′UTR of the two genes rather than by the promoter. Moreover, treatment with cycloheximide inhibited the compensatory regulation. Our results indicate a novel regulation mechanism of HNRNPA1 and A2 expression. Through compensatory regulation, the expression levels of HNRNPA1 and HNRNPA2 are strictly controlled within a certain range to maintain normal cellular activities under different physiological conditions.

## 1. Introduction

Heterogeneous nuclear ribonucleoprotein A1 (HNRNPA1, also known as UP1) and HNRNPA2 (also known as A2B1), encoded by different genes, are among the most abundant RNA-binding proteins and nucleoplasm shuttle proteins. Both HNRNPA1 and HNRNPA2 can produce multiple transcripts through alternative splicing. Full-length HNRNPA1 contains 11 exons and encodes 372 amino acids (HNRNPA1b, 38 kDa), whereas a shortened transcript produced by exon 7b skipping contains 320 amino acids (lacking amino acids 253–303; HNRNPA1a, 34 kDa). Full-length HNRNPA2 contains 12 exons, and produces four proteins due to alternative splicing of exon 2 and exon 9, which contain HNRNPB1 (353 amino acids, 38 kDa), HNRNPA2 (341 amino acids, 36 kDa), HNRNPB1b (313 amino acids, 33 kDa), and HNRNPA2b (301 amino acids, 31 kDa) (He and Smith, 2009; Han et al., 2010). In addition, a large number of pseudogenes of the two genes are distributed throughout the genome. In cells from different tissues, HNRNPA1a and HNRNPA2 are the main executors of HNRNPA1 and HNRNPA2 gene function, as they are expressed at much higher levels compared to the other transcripts.

HNRNPA1 and HNRNPA2 share a similar structure composed of two RNA recognition motifs (RRMs) and one glycine-rich domain (GRD). The RRM-glycine series structure can bind to nucleic acids and proteins, and thus participates in multiple levels of nucleic acid metabolism and transport, including alternative splicing (Mayeda et al., 1994; Martinez-Contreras et al., 2007), RNA stability (Fahling et al., 2006; Zhao et al., 2009), RNA transport (Rebane et al., 2004; Carson and Barbarese, 2005), telomere repair (Moran-Jones et al., 2005; Zhang et al., 2006), and transcription and translation regulation (Campillos et al., 2003; Zhao et al., 2008; Lin et al., 2009). Embryos of homozygous mice in which HNRNPA1 has been knocked out do not survive, whereas the embryos of heterozygous mice exhibit numerous changes with alternative splicing and gene expression accompanied by severe muscular development defects, suggesting that HNRNPA1 play indispensable roles in the development process (Liu et al., 2017).

HNRNPA1 and HNRNPA2 have also been associated with the occurrence of many human diseases. In particular, they are abnormally expressed in various types of tumors such as small cell lung cancer and gastric cancer (Romero-Garcia et al., 2014; Chen et al., 2018), and knockdown of HNRNPA1 or the use of adapters targeting HNRNPA2 can inhibit cancer cell proliferation, suggesting potential targets for cancer treatment (Li et al., 2015; Liu et al., 2016). Genetic studies have shown that a single amino acid mutation in the GRD domain of HNRNPA1 and HNRNPA2 protein is related to the proteinopathy of multiple systems and amyotrophic lateral sclerosis (Kim et al., 2013). In addition, HNRNPA1 and HNRNPA2 play an important role in RNA metabolism, which is consistently dysregulated in neurodegenerative diseases. Indeed, changes of HNRNPA1 and HNRNPA2 expression can improve the condition of patients with amyotrophic lateral sclerosis, spinal muscular atrophy, Alzheimer’s disease, and other neurodegenerative diseases (Bekenstein and Soreq, 2013). Therefore, exploring the mechanism underlying the expression regulation of HNRNPA1 and HNRNPA2 may improve the understanding of these vital cellular processes and diseases. Toward this end, in the present study, we evaluated the compensatory expression and regulation of HNRNPA1 and HNRNPA2, and performed preliminary exploration of the molecular mechanism.

## 2. Materials and methods

### 2.1. Protein structural analysis and sequence alignment 

The family and domains options in the UniProt databaseUniProt Consortium (2021). UniProt database [online]. Website https://www.uniprot.org/ [accessed 00 Month Year]. were used for protein structure analysis. The full-length transcripts of HNRNPA1b (NM_031157.3, NP_112420.1) and HNRNPA2 (NM_031243.2, NP_112533.1) were obtained from the National Center of Biotechnology Information, and the HNRNPA1 and HNRNPA2 protein sequences were aligned with Clustal Omega.EMBL-EBI (2021). Clustal Omega [online]. Website https://www.ebi.ac.uk/Tools/msa/clustalo/ [accessed 00 Month Year].

### 2.2. Plasmid construction and siRNAs

For overexpression, the previously generated plasmids pCGT7-HNRNPA1 and pCGT7-HNRNPA2 containing an N-terminal T7-tag (T7-A1 and T7-A2, respectively), referenced by (Hua et al., 2008). PGL3-A1/2 was constructed from two fragments: a 1084-bp fragment of the A1 promoter containing 1078 bases upstream of the start codon and the first two amino acids, and a 1323-bp fragment of the A2 promoter containing 1317 bases upstream of the start codon and the first two amino acids. These fragments were cloned into PGL3-basic vector (Promega, Madison, WI, USA) using *Mlu I* and *Xho I* digestion sites. To generate plasmid pmiR-A1/2, *Sal I* and *Not I* restriction sites were added to both ends of the HNRNPA1 and HNRNPA2 3′UTR, and subcloned into the *Xho I/Not I* sites of the pmiR-RB-Reporter vector (RiboBio, Guangzhou, China). Restriction endonucleases were purchased from New England Biolabs (Beverly, MA, USA). Primer pairs used for cloning are listed in supplementary table. All siRNAs were purchased from Genepharma (Shanghai, China), and their sequences are given in Table S1. 

### 2.3. Cell culture and transfection

HEK293 cells (Cell Bank, Chinese Academy of Sciences, Shanghai, China) were cultured in Dulbecco’s modified Eagle’s medium (Invitrogen, Thermo Fisher Scientific, Waltham, MA, USA), supplemented with 10% (v/v) fetal bovine serum (Gibco, Thermo Fisher Scientific), 100 U/mL penicillin, and 100 μg/mL streptomycin (Beyotime Biotechnology, Shanghai, China) at 37 °C in a humidified 5% CO2 atmosphere. Cells in the logarithmic growth phase were seeded in a 6-well plate at a density of 105 cells/well. The next day, 1 μg of plasmid or 100 nM siRNA was delivered to the cells using Lipofectamine 2000 (Life Technologies, Carlsbad, CA, USA) following the manufacturer instructions. RNAs and proteins were extracted at 48 h after transfection. The influence of each siRNA or plasmid on the expression of HNRNPA1 and HNRNPA2 at the mRNA and protein level was then detected by reverse transcription-quantitative polymerase chain reaction (RT-qPCR) and Western blotting, respectively.

### 2.4. Reverse transcription-polymerase chain reaction (RT-PCR)

Total RNA was extracted from the transfected cells using TRIzol reagent (Life Technologies), and 1 μg of each RNA sample was used in a 20-μl reaction for first-strand cDNA synthesis with oligo (dT)18 and M-MLV reverse transcriptase (HiScript II Q Select RT SuperMix, Vazyme Biotech, Nanjing, China). The products were amplified semiquantitatively using 28 cycles (95 °C for 15 s, 58 °C for 15 s, 72 °C for 40 s) with a series of primers (Table S2). PCR products were separated by agarose gels, and transcripts were quantified using ChamQ Universal SYBR qPCR Master Mix kit (Vazyme Biotech) on an ABI 7500 fluorescent quantitative PCR instrument using gene-specific primers (Table S3) according to the manufacturer instructions.

### 2.5. Western blotting

The transfected cells were harvested in lysis buffer (Beyotime Biotechnology) to obtain the total protein. Protein samples were separated by 10% SDS-PAGE and electroblotted onto polyvinylidene fluoride membranes (Millipore, Burlington, MA, USA). The membranes were blocked for 2 h with 5% (w/v) nonfat milk. The blots were then probed with monoclonal antibodies (anti-T7 antibody, Sigma-Aldrich, St. Louis, MO, USA; anti-β-tubulin antibody, Santa Cruz Biotechnology, Dallas, TX, USA; 1:1000 dilution) or polyclonal antibodies (anti-HNRNPA1 antibody, Proteintech, Rosemont, IL, USA; anti-HNRNPA2 antibody, Proteintech**, **1:1000 dilution) overnight at 4 °C followed by incubation with secondary IRDye 680RD goat antimouse or goat antirabbit antibody (LI-COR Biosciences, Lincoln, NE, USA; 1:2000 dilution). Protein signals were detected with an Odyssey Infrared Imaging System (LI-COR Biosciences). ImageJ software was used for gray intensity analysis.

### 2.6. Dual-luciferase reporter assay

The pmiR-RB-Report vector was used as a dual-luciferase reporter plasmid. Since the PGL3 vector only has the firefly luciferase report gene, the pRL-TK plasmid (Promega) was used as a transfection control and cotransfected with PGL3. The transfected cells were washed with phosphate-buffered saline and the fluorescence activity was detected using a luciferase analysis kit (Promega). In brief, the cells were lysed with 1× PLB lysate, and 15 μL of the PLB lysate supernatant was added to each well of a new 96-well optical plate, mixed with 100 μL LAR II containing luciferase, and then 100 μL Stop & Glo reagent was added for detection of the luminescence intensity of *Renilla *luciferase, and corresponding ratios were calculated.

### 2.7. Statistical analysis

SPSS17.0 was used for statistical analyses (SPSS Inc., Chicago, IL, USA). Student’s t-test was used to analyze the differences between two groups, and the data are presented as the mean ± SEM; p < 0.05 was considered statistically significant. GraphPad Prism was used to draw the bar chart.

## 3. Results

### 3.1. High homology between HNRNPA1 and HNRNPA2

**Figure 1:**
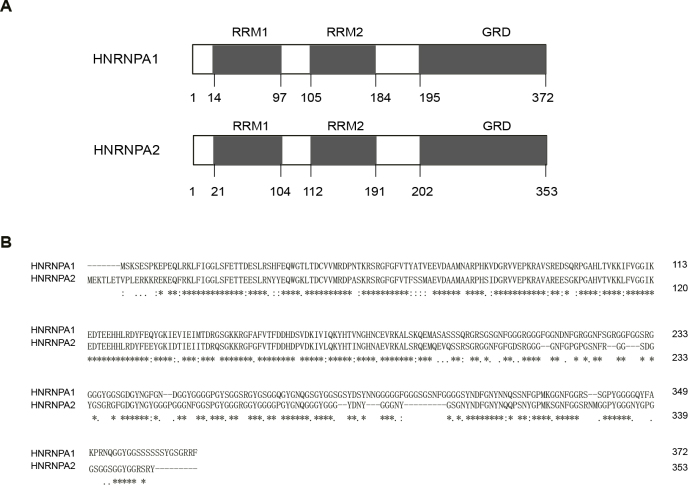
High homology of HNRNPA1 and HNRNPA2. (A) HNRNPA1 and HNRNPA2 primary sequence of the peptides. The number represents the location of amino acids, and the gray area represents the location of functional domains. (B) Amino acid sequence alignment of HNRNPA1 and HNRNPA2. * indicates an identical amino acid at that site.

The publicly available full-length HNRNPA1 and HNRNPA2 amino acid sequences were retrieved and compared. Both proteins contain three domains: RRM1, RRM2, and GRD (Figure 1A). Sequence alignment showed that overall amino acid sequence homology of 64% (238/372), with 78% (65/83) homology for the RRM1 domain, 86% (68/79) for the RRM2 domain, and 61% (92/151) for the GRD domain (Figure 1B). Peptide sequences comparison indicated that HNRNPA1 and HNRNPA2 are highly homologous, and the higher homology in their RRM domains suggests similar functions in regulating RNA metabolism.

### 3.2. HNRNPA1 and HNRNPA2 are paralogs that compensatory regulate each other’s expression

**Figure 2:**
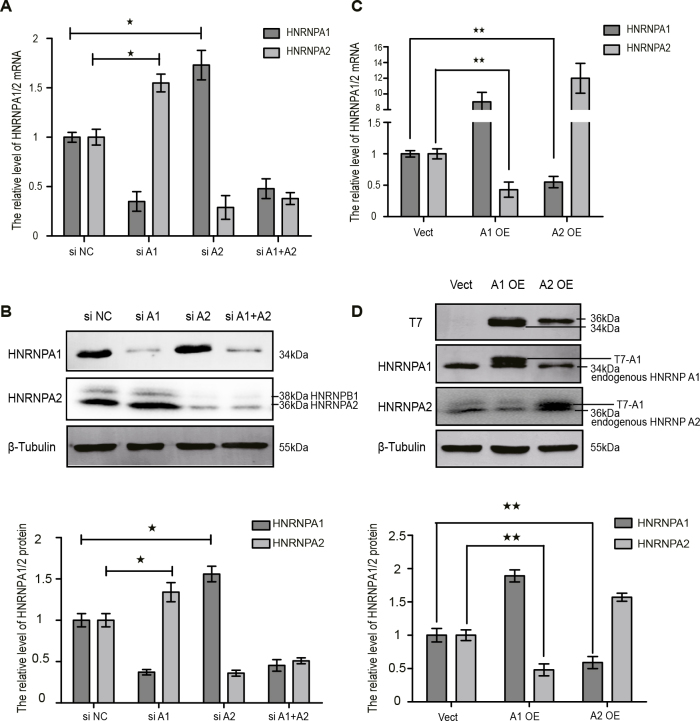
HNRNPA1 and HNRNPA2 are paralogs that compensate for each other’s expression. (A and C) Histograms showing the mRNA levels of HNRNPA1 and HNRNPA2 analyzed by RT-qPCR; data were normalized to the level of GAPDH. (B and D) Protein levels of HNRNPA1 and HNRNPA2 analyzed by Western blot; data were normalized to β-tubulin. The statistical results from three independent repeated experiments, compared with siNC or empty vector groups, * p < 0.05, ** p < 0.01.

RT-qPCR data showed that HNRNPA1 siRNA caused a 65% decrease in the HNRNPA1 expression level compared with NC siRNA, but upregulated the mRNA level of HNRNPA2 by 0.55-fold (Figure 2A). Similarly, HNRNPA2 siRNA caused a 71% reduction in the level of HNRNPA2 expression, but upregulated HNRNPA1 expression by 0.73-fold. Simultaneous transfection of HNRNPA1 siRNA and HNRNPA2 siRNA resulted in a 52% and 62% decrease of HNRNPA1 and HNRNPA2 expression, respectively, showing less effective silencing than transfection with a single siRNA. Western blotting showed consistent results at the protein level as observed with qPCR at the mRNA level. The HNRNPA1 or HNRNPA2 protein level was effectively knocked down, and a significant increase of HNRNPA1 or HNRNPA2 expression was observed (0.34-fold and 0.56-fold increase, respectively) (Figure 2B).

In addition, overexpression of HNRNPA1 and HNRNPA2 markedly decreased the mRNA level of HNRNPA2 and HNRNPA1*,* respectively (Figure 2C). Western blotting showed consistent results, with a 0.9-fold increase in the protein expression of HNRNPA1 after transfection of the T7-A1 plasmid, whereas the level of HNRNPA2 protein was decreased by 52%. Similarly, the T7-A2 plasmid increased the HNRNPA2 expression level by 0.6-fold and caused a 41% reduction of the HNRNPA1 expression level (Figure 2D).

These results suggested the compensatory regulation of expression between HNRNPA1 and HNRNPA2. Further, the consistency between the RT-qPCR and Western blotting results showed that changes in the two genes were consistent at the mRNA and protein levels, suggesting that the compensatory regulation occurred at the transcriptional or posttranscriptional process of gene expression rather than at the translation level.

Regulating gene expression in eukaryotes generally includes three levels: transcription, posttranscription (alternative splicing, RNA stability, etc.), and translation. In the following, we discuss the mechanism of compensatory regulation from three levels of alternative splicing, RNA stability, and translation. 

### 3.3. Compensatory regulation of HNRNPA1 and HNRNPA2 is not mediated by alternative splicing

**Figure 3:**
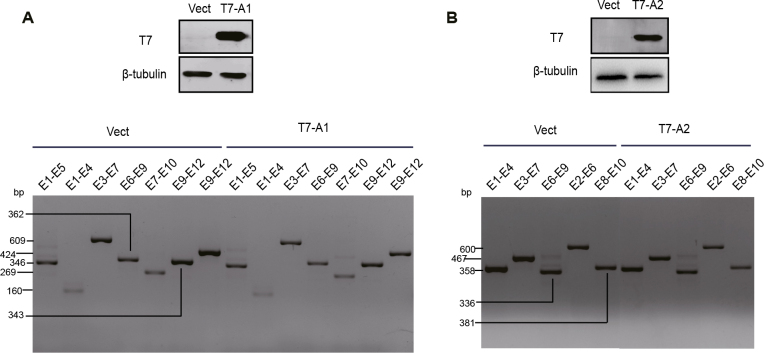
Effect of HNRNPA1 and HNRNPA2 on alternative splicing of HNRNPA2/A1. (A) Effect of HNRNPA1 overexpression on HNRNPA2 splicing. (B) Effect of HNRNPA2 overexpression on HNRNPA1 splicing. The transfected cells were split in two parts for Western blot and RT-PCR, respectively. A monoclonal antibody against the T7 tag was used to test the efficacy of HNRNPA1 and HNRNPA2 overexpression, and β-tubulin antibody was used as a loading control. Numbers on the left of the graph represent the length of the PCR products. “E1–E5” indicate the binding site of the forward and reverse primer in exon 1 and exon 5 of HNRNPA1, respectively.

The most common function of HNRNPA1 and HNRNPA2 is in the regulation of alternative splicing. It has been reported that HNRNPA1 regulates its own expression by inhibition of intron 10 splicing of HNRNPA1 pre-mRNA (Suzuki and Matsuoka, 2017). Therefore, we hypothesized that the compensatory regulation between HNRNPA1 and HNRNPA2 may be mediated by alternative splicing. To test this hypothesis, several primers were used to examine alternative splicing by the endogenous HNRNPA1 and HNRNPA2 gene in HEK293 cells that cover all exons of the two genes. Using the cells transfected with pCGT7 empty vector as a control, overexpression of HNRNPA1 with T7-A1 plasmid did not substantially affect alternative splicing except for a slight difference in exon splicing of Exon 7 and Exon 10 on HNRNPA2 (Figure 3A). Overexpression of HNRNPA2 with T7-A2 plasmid did not significantly alter the alternative splicing of HNRNPA1 (Figure 3B). These results suggested that alternative splicing is not the main cause of the compensatory expression regulation of HNRNPA1 and HNRNPA2. Interestingly, overexpression of HNRNPA1 or HNRNPA2 decreased the production of each primer pair compared with the control group, supporting that the hypothesis of the compensatory regulation was the change in total RNA rather than a change in alternative splicing of some exons or introns.

### 3.4. Compensatory regulation of HNRNPA1 and HNRNPA2 is mediated by their 3′UTRs 

**Figure 4:**
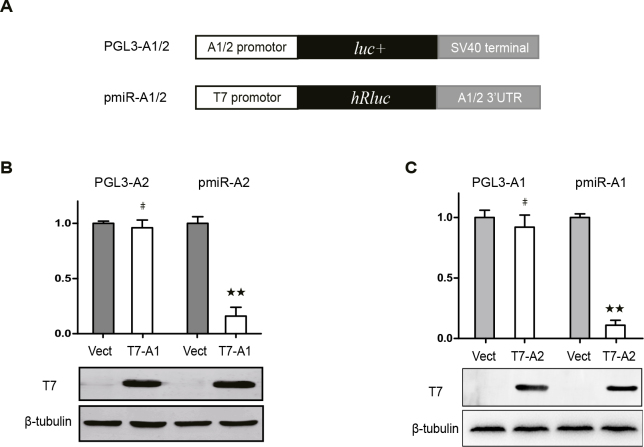
Compensatory regulation of HNRNPA1 and HNRNPA2 was mediated by their 3′UTRs. (A) Diagram of the construction of the luciferase reporting system. (B and C) Effects of HNRNPA1 and HNRNPA2 overexpression on activity of the HNRNPA1 and HNRNPA2 promoter and 3’UTR. A monoclonal antibody against the T7 tag was used to test the efficacy of HNRNPA1 and HNRNPA2 overexpression, and β-tubulin antibody was used as a loading control. Compared with empty vector, ** p < 0.01, # p > 0.05, n = 3.

Regulation mechanisms at the RNA level include transcription, splicing, and RNA stability. Two luciferase reporters were used to test molecular mechanism of compensatory regulation with respect to transcription and RNA stability. The HNRNPA1 and HNRNPA2 promoter region was cloned into the PGL3 vector to construct the reporter plasmid PGL3-A1/A2, and the HNRNPA1 and HNRNPA2 3′UTR sequence was cloned into the vector pmiR-RB-Report to construct the plasmid pmiR-A1/A2 (Figure 4A). The luciferase reporter plasmids were then cotransfected with the T7 empty control vector or T7-A1/A2 plasmid, and luciferase activity in each group was detected 48 h posttransfection. Overexpression of HNRNPA1 did not affect luciferase activity of pmiR-A2 (HNRNPA2 promoter) but significantly inhibited the luciferase activity of PGL3-A2 (HNRNPA2 3′UTR) (Figure 4B). For the impact of HNRNPA2 protein on HNRNPA1 expression, the results showed that overexpression of HNRNPA2 did not affect luciferase activity of pmiR-A1 (HNRNPA1 promoter) but significantly inhibited the luciferase activity of PGL3-A1 (HNRNPA1 3′UTR) (Figure 4C). These results suggested that the compensatory regulation of HNRNPA1 and HNRNPA2 was mediated by their 3′UTRs rather than by their promoters.

### 3.5. Compensatory regulation of HNRNPA1 and HNRNPA2 is inhibited by cycloheximide

**Figure 5:**
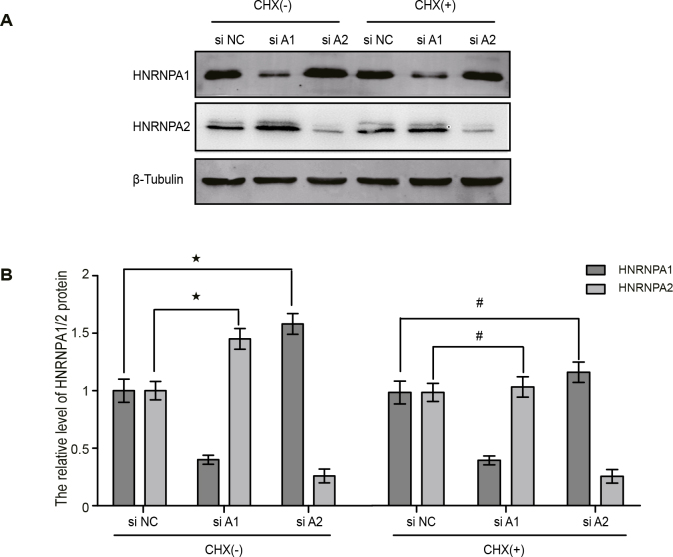
Compensatory degradation of HNRNPA1 and HNRNPA2 was mediated by the NMD pathway. (A) Western blot analysis of the protein expression of HNRNPA1 and HNRNPA2 after the transfected cells were treated with CHX. (B) Quantification of HNRNPA1 and HNRNPA2 expression in (A). The data were normalized to β-tubulin. * p < 0.05, # p > 0.05 versus siNC group, n = 3.

Cycloheximide (CHX) is a compound that inhibits the protein biosynthesis process of eukaryotes and is also an inhibitor of nonsense-mediated RNA decay (NMD). We next tested whether CHX plays a role in compensatory regulation of HNRNPA1 and HNRNPA2. The HEK 293 cells were treated with 75 μM CHX after 30 h transfection of siRNA, and HNRNPA1 and HNRNPA2 expression was detected by Western blotting 18 h after CHX treatment. Knockdown of HNRNPA1 and HNRNPA2 did not significantly increase the expression of HNRNPA2/A1 in cells treated with CHX compared with the no-CHX group (Figures 5A and 5B). These results suggest that compensatory regulation of HNRNPA1 and HNRNPA2 is inhibited by CHX, the mechanism may be the inhibition of extensive protein synthesis or NMD.

## 4. Discussion

A disorder in gene expression can result in disease development; thus, gene expression is regulated at several levels through many mechanisms, including the transcription, posttranscription, translation, and posttranslation levels. HNRNPA1 and HNRNPA2, as abundant RNA-binding proteins are well-known to widely regulate gene expression; however, the mechanisms by which their own expression is regulated have thus far remained unclear. Here, we reported the compensatory regulation of HNRNPA1 and HNRNPA2, and confirmed that they are mediated by their respective 3′UTRs. 

There are numerous pseudogenes of HNRNPA1 and HNRNPA2 distributed throughout the genome. Although we strictly paid attention to the specificity of primers during the experiment, we ignored the influence of pseudogene expression mRNA on the results of RT-PCR and real time PCR. Fortunately, the experiment results of western blot and luciferase reporters were not affected by this. Therefore, the existence of pseudogenes does not affect our key conclusions.

Wang et al. (2017) reported a degradation mechanism of HNRNPA1 in which epidermal growth factor promoted its degradation by activating ubiquitin signaling. In the present study, we found that the change in the protein expression of HNRNPA1 and HNRNPA2 was consistent with that of its mRNA, suggesting expression regulation by more than one mechanism. Indeed, the cooperation of multiple mechanisms ensures that the gene expression regulation of eukaryotes is complex and precise. Overexpression of several RNA-binding proteins has been reported to provide feedback to regulate their own expression through self-regulation (Sun et al., 2010; Dai et al., 2012; Suzuki and Matsuoka, 2017); however, self-regulation pathway does not apply to all RNA binding proteins. To our knowledge, the compensatory regulation of homologous RNA-binding proteins has not been reported until now. 

Sun et al. (2010) reported the self-regulation mechanisms of SRSF1, a member of the arginine serine family. At the posttranscriptional level, SRSF1 overexpression regulated the alternative splicing of its own endogenous genes and induced the production of new unstable transcripts, which were then degraded by the NMD pathway through its 3′UTR. At the translation level, SRSF1 overexpression altered the distribution of its mRNA by inducing the transformation to a monosome, thus inhibiting RNA translation. Although these RNA-binding proteins also exhibit self-regulation, the mechanisms are quite different to that identified for HNRNPA1. Suzuki and Matsuoka (2017) reported the autoregulation of HNRNPA1 as exogenous overexpression of HNRNPA1 downregulated the expression of endogenous HNRNPA1. In this case, the autoregulation of HNRNPA1 neither depended on its 3′UTR nor occurred through the NMD pathway, but was rather caused by inhibition of the splicing of intron 10 in endogenous pre-mRNA mediated by HNRNPA1 itself. However, in this study, we did not detect a change of alternative splicing of HNRNPA1 intron 10 caused by HNRNPA2 overexpression. Alternatively, we found that the compensatory regulation of HNRNPA1 and HNRNPA2 expression involved the 3′UTR. This differs from the self-regulation mechanism of HNRNPA1, indicating that HNRNPA1 and HNRNPA2 expression are controlled by complex and diverse mechanisms.

The detailed molecular mechanism by which HNRNPA1 and HNRNPA2 proteins activate the HNRNPA1 and HNRNPA2 mRNA NMD pathway requires further examination. The termination codon of both genes is located in the penultimate exon. Regions downstream of the termination codon contain the exon junction complex, which can stimulate the rapid deadenylation of mRNA and degrade mRNA by the CCR4-Not complex. Geissler et al. (2016) reported that UAASUAUU, a conserved sequence in the 3′UTR, is widely involved in RNA degradation, which can bind to HNRNPA1 and HNRNPA2 to recruit the CCR4-Not complex, thereby initiating target RNA degradation. Although bioinformatics analysis showed that the optimal binding sequence of HNRNPA1 and HNRNPA2 protein was UAGGGU/A (Burd and Dreyfuss, 1994; Bruun et al., 2016), the high abundance of these proteins in cells may enable binding to other sequences. The 3′UTRs of HNRNPA1 and HNRNPA2 contain multiple SUUAU motifs reported by Geissler et al. (2016) and the optimal UAGG motif. Therefore, the mechanism of HNRNPA1 and HNRNPA2 compensatory regulation may involve direct binding of HNRNPA1 and HNRNPA2 to the HNRNPA1 and HNRNPA2 mRNA 3′UTR to promote the recruitment of the CCR4-Not complex after NMD activation. When the HNRNPA1 and HNRNPA2 protein concentration changes, the number of CCR4-Not complexes recruited by HNRNPA1 and HNRNPA2 protein will change accordingly, altering the degradation rate of mRNA and enabling compensatory regulation.

HNRNPA1 and HNRNPA2 have very similar functions, and few studies have directly demonstrated their functional differences. Therefore, further investigation is warranted to determine why cells would spend the energy to express two proteins with the same functions at such high levels. The compensatory regulation phenomenon found in this study is similar to the widely observed genetic compensation phenomenon as a common fault tolerant mechanism in eukaryotes. When a gene is mutated or deleted, a series of reactions occur to improve the expression of other similar genes in its family, thus compensating for the function of the inactivated gene. Because HNRNPA1 and HNRNPA2 play such a crucial role in regulating gene expression, its abnormal expression can cause numerous downstream gene expression changes. Through compensatory regulation, HNRNPA1 and HNRNPA2 expression can be buffered in a complex and changeable environment to ensure the normal progression of various life activities.

## Funding

This work was supported by Natural Science Foundation of Jiangsu Province (BK20160418) and Municipal Health Commission of Nantong (grant no: MA2020019).

## Data availability statement

The datasets analyzed during the current study are available from the corresponding author on reasonable request.

Supplementary Materials
