# Supplementary material for: Compensatory expression regulation of highly homologous proteins HNRNPA1 and HNRNPA2
Source: Turk J Biol. 2021 Apr 20;45(2):187–95. doi: 10.3906/biy-2010-29 (PMC8068773; doi:10.3906/biy-2010-29)
Supplement: Supplementary file 1 — Supplementary Materials [file turkjbio-45-187-sup001.pdf]

Supplementary tables

Table S1. Primers for cloning luciferase report vector.

Names or products	Forward primer (from 5′ to 3′)	Reverse primer (from 5′ to 3′)
A1 promotor	acacgcgtTCTCCCACATTCCGATGGCC	atctcgagAGACATGACGGCAGGGTG
A2 promotor	acacgcgtACGCGTGTGGCATCTGAAGCAC	atctcgagCTCCATCGCGGACTCAGTCG
A1 3′UTR	acgtcgacTAATTAGGAAACAAAGCTTAGCAGG	acgcggccgcTTCAAGAGAATTAAATCGTTTATTG
A2 3′UTR	acgtcgacTGAGCTTCTTCCTATTTGCC	acgcggccgcTCCTTTAGAATTATTTATTAAATCATAAATG

Table S2. Primers for detecting gene mRNA level.

Gene name	Forward primer (from 5′ to 3′)	Reverse primer (from 5′ to 3′)
HNRNPA1	TCAGAGTCTCCTAAAGAGCCC	ACCTTGTGTGGCCTTGCAT
HNRNPA2	AGCTTTGAAACCACAGAAGAA	TTGATCTTTTGCTTGCAGGA
GAPDH	TCAACGACCACTTTGTCAAGCTCA	GCTGGTGGTCCAGGGGTCTTACT

Table S3. Sequences of siRNAs used to knock down HNRNPA1 and HNRNPA2 and NC siRNA.

Gene	Sense (from 5′ to 3′)
HNRNPA1 siRNA	CAGCUGAGGAAGCUCUUCA
HNRNPA2 siRNA	GGAACAGUUCCGUAAGCUC
NC siRNA	UUCUCCGAACGUGUCACGU

Table S4. Primers for RT-PCR to examine HNRNPA1 and HNRNPA2 alternative splicing.

Name	Primer (from 5′ to 3′)	Size (bp)
A2 E1-E5	F- ACTGAGTCCGCGATGGAG	346
	R-AGTATCTTCTTTAATTCCG	
A2 E1-E4	F- ACTGAGTCCGCGATGGAG	160
	R- TTGATCTTTTGCTTGCAGGA	
A2 E3-E7	F- AGCTTTGAAACCACAGAAGAA	609
	R- AGATCCTCCTCTAAAGTTACTTC	
A2 E6-E9	F- AGAAATACCATACCATCAATGG	362
	R- TCCTCCATAGTTGTCATAACCA	
A2 E7-E10	F- AGG CAA CTT TGG CTT TGG	269/389
	R-CTCCACCATATGGTCCC	
A2 E9-E12	F- TGGTTATGACAACTATGGAGGAG	343
	R- ACTGCATATTTATGCATGACTG	
A2 E9-E12-2	F- TGGTTATGACAACTATGGAGGAG	424
	R-ACAGTAAGGTAATGTTATTAAATAATCC	
A1 E1-E4	F- CCGTCATGTCTAAGTCAGAG	358
	R- CTTCAGTGTCTTCTTTAATGCC	
A1 E3-E7	F- CCAGAGAAGATTCTCAAAGACC	467
	R- TCCATTATAGCCATCCCCAC	
A1 E6-E9	F- ACTTTGGTGGTGGTCGTGG	336
	R-TACTGCTGCTGCTGGAACC	
A1 E2-E6	F- AGCTGAGGAAGCTCTTCATTG	600
	R-ACCGAAACCACCTCCACG	
A1 E8-E10	F- AGGTGGTGGAAGCTACAATGATTTTG	381
	R- AGTCACAAATACAGTCCTCGAG	
